# dSETDB1 and SU(VAR)3–9 Sequentially Function during Germline-Stem Cell Differentiation in *Drosophila melanogaster*

**DOI:** 10.1371/journal.pone.0002234

**Published:** 2008-05-21

**Authors:** Jeongheon Yoon, Kyu-Sun Lee, Jung Sun Park, Kweon Yu, Sang-Gi Paik, Yong-Kook Kang

**Affiliations:** 1 Center for Regenerative Medicine, Korean Research Institute of Bioscience and Biotechnology (KRIBB), Daejeon, Korea; 2 Department of Biology, Chungnam National University, Daejon, Korea; University of Munich and Center of Integrated Protein Science, Germany

## Abstract

Germline-stem cells (GSCs) produce gametes and are thus true “immortal stem cells”. In *Drosophila* ovaries, GSCs divide asymmetrically to produce daughter GSCs and cystoblasts, and the latter differentiate into germline cysts. Here we show that the histone-lysine methyltransferase dSETDB1, located in pericentric heterochromatin, catalyzes H3-K9 trimethylation in GSCs and their immediate descendants. As germline cysts differentiate into egg chambers, the dSETDB1 function is gradually taken over by another H3-K9-specific methyltransferase, SU(VAR)3–9. Loss-of-function mutations in *dsetdb1* or *Su(var)3–9* abolish both H3K9me3 and heterochromatin protein-1 (HP1) signals from the anterior germarium and the developing egg chambers, respectively, and cause localization of H3K9me3 away from DNA-dense regions in most posterior germarium cells. These results indicate that dSETDB1 and SU(VAR)3–9 act together with distinct roles during oogenesis, with dsetdb1 being of particular importance due to its GSC-specific function and more severe mutant phenotype.

## Introduction

*Drosophila* oogenesis is a complex developmental process involving the coordinated differentiation of germline and somatic cells, and begins with asymmetric division of a single germline stem cell (GSC) [1], [2]. This GSC is located at the tip of each ovariole in the germarium, which is a generative region that is divided into sub-regions such as region-1, -2a, -2b and -3. After each GSC division, the posterior daughter cell becomes a “cystoblast”, leaves region-1, undergoes four synchronous, incomplete divisions to form a 16-cell germline cyst [3], [4], and steadily moves in a posterior direction through the germarium. Of the 16 interconnected cells, one cell develops into the oocyte whereas the other 15 develop into polyploid nurse cells [5]. This 16-cell cyst becomes surrounded by a monolayer of follicle cells and buds off from the posterior germarium to form an egg chamber [6], [7], which ultimately gives rise to a single mature oocyte ready for fertilization.

The germline cells, including the GSCs are the only population from which both parental epigenetic information and genetic information can be transferred to progeny. This indicates that, other than the known pluripotency [8], the germline cells possess another important property - an exceptional capacity for epigenetic modifications of the genome [9]. In fact, germ-cell development is associated with a dynamic process of epigenetic reprogramming, leading to re-construction of the whole genome-level epigenetic state [9], [10], [11], [12], [13]. The developmental significance of this has driven studies to investigate the epigenetic changes occurring in the germline cells. Therefore, germ-cell development is an excellent system to study how the epigenetic system involving DNA methylation and histone lysine methylation is erased, re-established, and maintained in the germ cells at the genome-wide level.

Histone-lysine methylation, which mainly occurs in the tails of histones H3 and H4, plays a pivotal role in cellular processes including heterochromatin formation, X-chromosome inactivation, and transcription regulation [14]. Lysine methylation is of particular interest because it can modulate the chromatin structure to a compacted state or a relaxed one, depending on which lysine residues are methylated. With regard to heterochromatin formation, histone H3 trimethylated at lysine 9 (H3K9me3) is enriched in pericentric heterochromatin and thereby recognized as typical of a heterochromatin marker [15], [16], [17], [18], [19]. Correct formation of heterochromatin is essential for chromosome stability and integrity, and is required for the proper segregation of chromosomes during mitosis [20] and the recombination events in fission yeast [21], which further demonstrates the biological significance of H3K9me3 that participates in heterochromatin formation. So far, several histone-lysine methyltransferases (HKMTases) with specificity to H3-K9 residues have been identified [14]. Some of them are implicated in germ-cell development. Male germ cells in mice lacking suv39h, which synthesizes H3K9me3 in pericentric heterochromatin, display severely impaired viability and chromosomal instability [22]. Mutant mice in which G9a is specifically inactivated in the germ-cell lineage exhibited a marked loss of mature sperm and oocytes [23].

In *Drosophila*, as in other organisms, H3-K9 methylation is associated with heterochromatin formation and gene silencing, and is catalyzed by multiple HKMTases including SU(VAR)3-9 [24], dG9a [25] and dSETDB1 (also known as *dmsetdb1* and *egg*) [26], [27], [28]. Unlike mammalian suv39h, *Su(var)3-9* mutants are viable, fertile, and morphologically indistinguishable from the wild-type flies, and so far have not been associated with defects in germ-cell development in *Drosophila* (Tschiersch et al., 1994). In addition, *dG9a* is abundantly expressed in the gonads of both sexes [29], and female mutants for dG9a are also fertile [25]. Even the *dG9a* and *Su(var)3-9* double mutants, *dG9a^13414^/dG9a^13414^; Su(var)3-9^06^/Su(var)3-9^06^*, can produce mature oocytes and are therefore fertile; although the proportion of larvae that survive the pupal stage is greatly reduced [25]. Therefore, in *Drosophila*, both *Su(var)3-9* and *dG9a* are expected to have less of a critical role in germ-cell development than in mammals.

Until recently, mammalian *SETDB1/Eset* has not shown a function related to germ-cell development. However, its *Drosophila* counterpart, *dsetdb1*, is closely associated with germ-cell development. *dsetdb1* homozygous mutants were shown to have degenerated egg chambers, and therefore be sterile [28]. SETDB1/Eset, like G9a, functions in euchromatic DNA regions by forming complexes with various transcription factors including KAP1 [30], [31]. Our previous results have demonstrated that homozygous mutation of *Eset* leads to peri-implantation lethality [32], indicating that *Eset* is necessary for early mammalian development. Recent observations in *Drosophila* demonstrated that dsetdb1 localizes mainly to the whole fourth chromosome [27], and shows H3-K9 methylation activity and transcriptional repression, both of which are specific to the fourth chromosome in the polytene chromosomes of the salivary glands [26], [27]. These results indicate that *dsetdb1* has functions and plays distinct roles in different tissues in *Drosophila*.

Germ-cell development ultimately generates the haploid cells that are responsible for the maintenance of the species. Understanding how epigenetic patterns are established in the germ line may eventually help in the prevention of heritable diseases, improvement of assisted reproductive technologies, and stem cell therapy [10]. As a trial to determine the molecular processes of germ-cell development, we investigated the roles of HKMTases in the *Drosophila* oogenesis. We found that dSETDB1, localized to pericentric heterochromatin in germ cells including the oocytes, is the only HKMTase that synthesizes trimethylated H3-K9 in the germ-cell lineages in the germarium, and this function is transferred to SU(VAR)3-9 in later oogenesis. Our results indicate that dSETDB1 is necessary for *Drosophila* oogenesis, and suggest that dSETDB1 has a role in coordinating the chromosomal integrity in the germ-cell lineages, and that loss of *dsetdb1* function leads to dysregulation of chromosome organization.

## Results

### Mutations in dsetdb1 gene cause severe defects in the ovary

**Figure 1:**
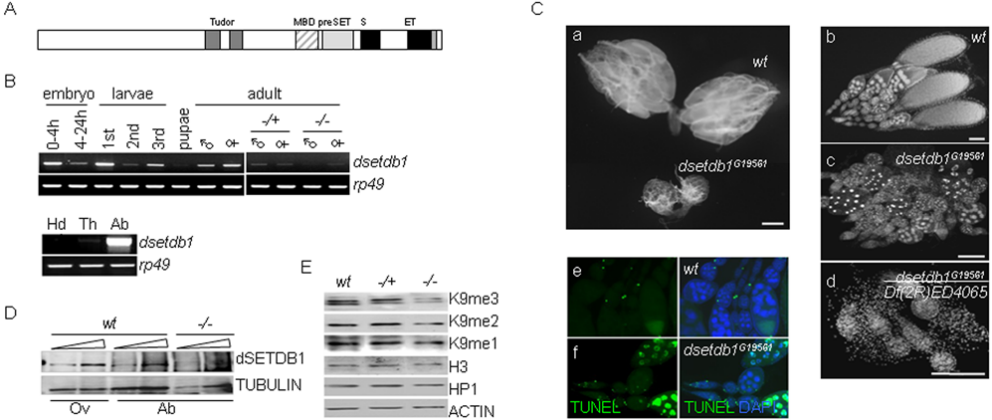
Expression of *dsetdb1 in Drosophila melanogaster*. (A) Schematic illustration of the dSETDB1 structure. MBD, methyl-binding domain; S and ET, bifurcated SET domain. (B) The *dsetdb1* transcript levels during *Drosophila* development (upper panel), or in female body segments (lower panel). ♂, male; ♀, female; −/+, heterozygotes (n = 15); −/−, homozygotes (n = 15); Hd, head; Th, thorax; Ab, abdomen. *Rp49,* loading control. (C) Apoptotic phenotypes of *dsetdb1^G19561^* ovaries. Ovaries isolated from wild-type and *dsetdb1^G19561^* females (a). Note the difference in size between wild-type and *dsetdb1* mutant ovaries. (b–d) Ovarioles from wild-type (b), *dsetdb1^G19561^* (c), and *dsetdb1^G19561^/Df(2R)ED4065* (d) flies. Homozygote females were unable to lay eggs, and were thus sterile; beyond stage 8 (in *dsetdb1^G19561^*), or in some cases at even earlier stages (*dsetdb1^G19561^/Df(2R)ED4065*), egg chambers were undetectable. (e–f) TUNEL assay. Most egg chambers were TUNEL-positive in *dsetdb1^G19561^* flies. Images were taken using a Carl Zeiss Axiovert 200M fluorescence microscope equipped with an ApoTome, or using an Olympus BX60 microscope. Scale bars, 1 mm in parts a and b, and 100 µm in parts c-e. (D) The protein levels of dSETDB1 in wild-type (*wt*) ovarian (Ov) and abdominal (Ab) protein extracts, and *dsetdb1^G19561^* abdominal extracts. α-TUBULIN, a loading control. (E) Western-blot analyses for H3K9me1, H3K9me2, H3K9me3, and HP1 in ovarian protein extracts. Histone H3 and β-ACTIN, loading controls. Wt, wild type; −/+, heterozygotes; −/−, homozygotes.

We studied on *dsetdb1* ([26], also named ‘*egg*’ [28] or ‘*dmsetdb1*’[27]) (Fig. 1A), a *Drosophila* homologue of human *SETDB1*. When the transcription level of *dsetdb1* was examined, a pattern of continuous oscillation during the life cycle was seen (Fig. 1B, upper panel). This result indicated that *dsetdb1* may have a vital role throughout *Drosophila* development. In the adult fly, *dsetdb1* was expressed slightly more in females than in males, and, in females, *dsetdb1* expression was predominantly in the abdominal area (Fig. 1B, lower panel).

We obtained a hypomorphic EP mutant [33], *dsetdb1^G19561^*, and a mutant (*dsetdb1^G19561^/Df(2R)ED4065)* transheterozygous for *dsetdb1^G19561^* and the chromosomal deficiency *Df(2R)ED4065* deleting the *dsetdb1* gene (deleted region, 60C8-60E7). The *dsetdb1* transcript was rarely detected in *dsetdb1* homozygous mutant flies (Fig. 1B). *Drosophila* with these mutants possessed ovaries of a markedly reduced size compared with wild-type flies (Fig. 1Ca). The *dsetdb1^G19561^/Df(2R)ED4065* mutant caused a slightly more-severe phenotype than the *dsetdb1^G19561^* mutant (Fig. 1Cb–d). Homozygous mutant females were unable to lay eggs, and were therefore sterile, which was in agreement with a previous report [28]. The *dsetdb1^G19561^* ovary carried egg chambers, but these were mostly degenerated in appearance, as demonstrated by analysis with the TUNEL assay (Fig. 1Ce–f). We raised an anti-dSETDB1 polyclonal antibody from rabbit. Western-blot analysis of abdominal protein extracts found only a negligible level, if any, of dSETDB1 in *dsetdb1^G19561^* mutants (Fig. 1D).

### dSETDB1 is primarily expressed in the germarium and is exclusively localized to DNA-dense regions of nuclei in the ovary

**Figure 2:**
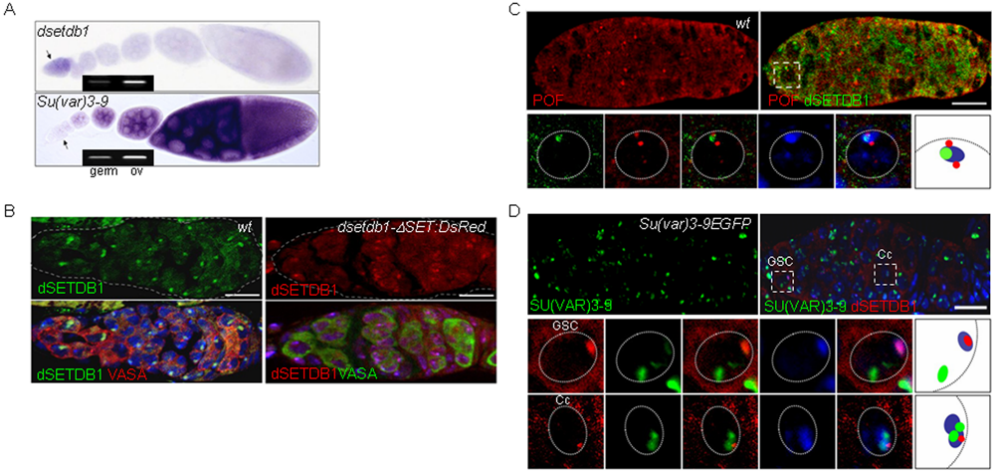
Pericentric heterochromatin localization of dSETDB1 in the germarium. (A) Whole-mount mRNA *in-situ* hybridization using a probe either for *dsetdb1* or *Su(var)3–9*. Arrows indicate the germaria. Germaria only were removed from the residual egg chambers and used for RT-PCR analyses (inset; germ, germaria sample; OV, ovarian sample). (B) Localization of endogenous (left panel) and exogenously expressed (right) dSETDB1 to pericentric heterochromatin in a wild-type and a transgenic *nanos-gal4>dsetdb1-ΔSET:DsRed2* germarium, respectively. The germarium was also stained for VASA, a germ-cell lineage marker, and counterstained for DNA, with 4′,6-diamidino-2-phenylindole (DAPI; blue). Dashed lines (gray) indicate boundaries of germaria. (C, D) The dSETDB1 signals are not co-localized with paint-of-fourth chromosome (POF, C) and SU(VAR)3-9-eGFP (D). Boxed areas are enlarged into individual channels or merged channels, together with the schematic illustration of the relative positions of individual signals (the right-most panel). In (D) GSC, germline-stem cell; Cc, cystocyte. Dotted circles are nuclear boundaries. Expression of SU(VAR)3-9-eGFP was induced by heat shock treatment. Scale bars, 10 µm.

We performed whole-mount RNA *in situ* hybridization (RISH) using a *dsetdb1* antisense probe. As shown in Fig. 2A, *dsetdb1* transcripts were mainly detected in the germarium, although not to a high level; these transcripts reappeared in the stage-10 egg chambers, most likely as maternal mRNA stock for embryonic development (data not shown). Analysis of expression of *Su(var)3-9*, the first and best characterized H3-K9-specific methyltransferase [34], in the ovary showed that it was highly expressed in developing egg chambers (Fig. 2A). When the RISH pattern of *dsetdb1* was paralleled with that of *Su(var)3-9*, a well-orchestrated, yin-yang expression pattern of *Su(var)3–9* and *dsetdb1* was evident. The RISH results simply show the main region of expression for each of *dsetdb1* and *Su(var)3-9* genes in the ovariole strings, and it does not necessarily mean that *dsetdb1* transcripts are more abundant in the germarium than *Su(var)3-9* transcripts. These results indicate that both *dsetdb1* and *Su(var)3-9* are expressed in the ovary and have roles in gametogenesis. On the other hand, *dG9a*, another H3-K9-specific methyltransferase, was shown to be mainly transcribed in stage-10 egg chambers, which indicates that the *dG9a* transcripts are mostly maternal stock for embryo development (Fig. S1). We isolated the germaria by separating them from stretches of developing egg chambers under a stereomicroscope. RT-PCR with germarial RNAs demonstrated that both *dsetdb1* and *Su(var)3-9* are expressed in the germarium (Fig. 2A inset).

**Figure 3:**
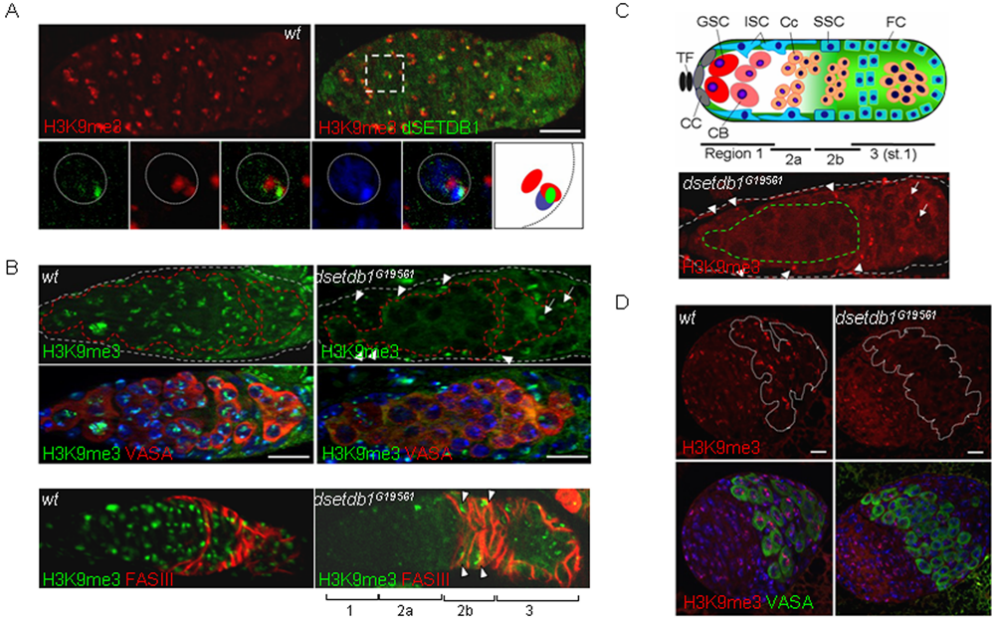
H3-K9 trimethylation in *dsetdb1^G19561^* ovaries is abolished in a germ-cell-specific manner. (A) Colocalization of H3K9me3 signals with dSETDB1 signals and DAPI-dense spots. A boxed area is enlarged into individual or merged channels, together with a schematic illustration of the relative positions of individual signals (the right-most panel). Dotted circles indicate nuclear boundaries. (B) Loss of H3K9me3 signals in the inner region of the anterior germarium (the ‘inner’ germarium) in the *dsetdb1^G19561^* germarium. Different cell populations in the germarium were stained either for VASA (upper panel) or Fas III (lower panel). (C) Diagram of the *Drosophila* germarium (upper panel). TF, terminal filament; CC, cap cell; GSC, germline-stem cell; ISC, inner-sheath cell; CB, cystoblast; Cc, cystocyte; SSC, somatic stem cell; FC, follicle cell. Subregions of the germarium (1, 2a, 2b, and 3) are indicated. Typical H3K9me3 pattern in a mutant germarium (lower panel), where the signal is absent from the inner germarium (dashed boundary in green; see text) but intact in the outer germarium (the region in between grey and green boundaries). (D) H3K9me3 signals are abolished in the VASA-positive primordial germ cells in the third-instar larval ovary of the *dsetdb1* mutant. Dashed lines (red in B and white in D) indicate the boundaries of VASA-positive cells. Germaria in A-C are outlined by dashed lines. The nuclei were counterstained with 4′,6-diamidino-2-phenylindole (DAPI; blue). Arrows and arrowheads in B (right panel) and C (lower panel) indicate H3K9me3 signals detected in the nurse cells and somatic cells of the outer germarium of the *dsetdb1* mutant. Scale bars, 10 µm.

Immunostaining of wild-type ovaries showed that dSETDB1 was particularly obvious in the germarium (Fig. 2B, left panel; see Fig. 3C for a schematic of the germarium structure). Interestingly, dSETDB1 signals were mainly detected in 4′,6-diamidino-2-phenylindole (DAPI)-dense, pericentric heterochromatic DNA regions in all germarium cells. This was unexpected because mammalian SETDB1/Eset is known to have a euchromatin-associated function [30] and also because Egg was expressed both in the cytoplasm and nucleus equivalently in the germarium [28]. The unusual heterochromatic localization of dSETDB1 was confirmed using a *dsetdb1-ΔSET:DsRed2* transgenic line in which *dsetdb1* expression was driven by a *nanos-gal4* promoter (Fig. 2B, right panel); DsRed2-dSETDB1 signals were localized to DAPI-dense regions in the transgenic germarium.

### dSETDB1 has distinct roles during Drosophila oogenesis, localizing at pericentric heterochromatin

Recent studies have reported that dSETDB1 is specifically localized to the entire fourth chromosome in the polytene chromosome spreads of the salivary glands [26], [27], which prompted us to immunostain the germarium for the painting of fourth chromosome (POF) [35] that is known to coat the entire fourth chromosome. When the germarium was doubly stained for dSETDB1 and POF, however, the POF signals, which were detected as one or two spots (reflecting the diploid genome) flanking the DAPI-dense region, were not localized with the dSETDB1 signals (Fig. 2C). This POF staining pattern was in agreement with a previous observation that POF signals decorating the pair of whole fourth chromosomes were seen as one or two clear foci in S2 and other somatic cells including ovarian follicle cells [36]. Since we identified the expression of *Su(var)3-9* in the germarium (Fig. 2A), we attempted to correlate dSETDB1 signals with SU(VAR)3-9 signals. Antibody specific to SU(VAR)3-9 was unavailable, so we used a GFP-tagged SU(VAR)3-9-expressing transgenic fly [24]. The main GFP-tagged SU(VAR)3-9 signals did not coincide with the dSETDB1 signals (Fig. 2D). This was especially true in the germarium region-1 and -2a (middle panels) where endogenous SU(VAR)3-9 is not thought to function (see below); however, in region-3 germarium (lower panels), in which the endogenous SU(VAR)3-9 is thought to function, both signals appeared to be largely overlapped (Fig. S2). Therefore, these findings indicate a peculiar function of dSETDB1 in the germarium.

The nuclei of would-be oocytes in posterior regions of the developing egg chambers had DAPI-dense spots, known as the karyosomes [37], at which strong dSETDB1 signals were identified (Fig. S3A), as reported recently [28]. Interestingly, ectopically expressed SU(VAR)3-9-eGFP signals were not detected in the karyosomes (Fig. S3B). It would be interesting to investigate the reason for the exclusion of SU(VAR)3-9-eGFP from the nuclei of would-be oocytes as the SU(VAR)3-9-eGFP appears in all egg chamber cells (data not shown). Taken together, these data indicate that dSETDB1, which is mainly located in the pericentric heterochromatin in the cells of the germarium and early egg chamber including would-be oocytes, presumably fulfills peculiar roles in the ovary. These roles differ from the known POF-associated function in the salivary glands [27] and SU(VAR)3-9 might not be a substitute for dSETDB1.

### dSETDB1 synthesizes trimethylated H3-K9 in the primordial germ cells and germ-line stem cells

Mammalian SETDB1/Eset [30], [32], [38], [39], like SU(VAR)3-9 [15], [22], [34], [40], synthesizes H3K9me3 [38]. Examination of a wild-type germarium showed that the major H3K9me3 signals were detected in DAPI-dense regions [16], [28] and, overall, were localized with the dSETDB1 signals (Fig. 3A). In the *dsetdb1^G19561^* mutant, however, H3K9me3 signals were absent from the inner part of the anterior germarium (Fig. 3B). As shown in Fig. 3C, for convenience of discussion, we divided the germarium into two parts: the ‘inner’ germarium consisting of VASA-positive cells [41] such as GSCs, cystoblasts and cystocytes in region-1 and region-2a; and the ‘outer’ germarium, which comprises the rest of the germarium, including region-2b and region-3 as well as a layer of the somatic cells surrounding region-1 and region-2a. The inner germarium cells of the *dsetdb1^G19561^* mutant lacked H3K9me3 signals, whereas the outer germarium cells maintained H3K9me3 signals (Fig. 3B, upper panel). When stained for Fasciclin III (Fas III), a marker for follicle and stalk cells derived from somatic stem cells [7], the mutant germarium was observed to have H3K9me3 signals crowded in Fas III-positive cells of region-2b and -3 germarium [42], [43] (Fig. 3B, lower panel). This phenotype was different from the previously reported one that used *egg^1473^* mutant flies to show that the whole germarium lost H3K9me3 signals [28], which reflects the severer phenotype of the *egg^1473^* mutant lacking the entire SET domain than our EP mutants. Representative H3K9me3 features in *dsetdb1^G19561^* mutant germarium are presented in Fig. 3C (lower panel). Examination of the third-instar larval ovary of mutants showed that VASA-positive primordial germ cells (PGCs) [44], which are precursors of the germline stem cells (GSCs) and normally express H3K9me3 signals (Fig. 3D, left panel), also lacked the H3K9me3 signals (right). The *in situ* database at Berkeley Drosophila Genome Project (BDGP; http://www.fruitfly.org.cgi-bin/ex/insitu.pl) site shows germ-cell- and gonad-specific *dsetdb1* expression in stage 13–16 embryos. These results demonstrate that *dsetdb1* functions in a germline-specific manner and, in the germarium of an adult ovary, trimethylates H3-K9 residues of GSCs and their early progeny cells.

**Figure 4:**
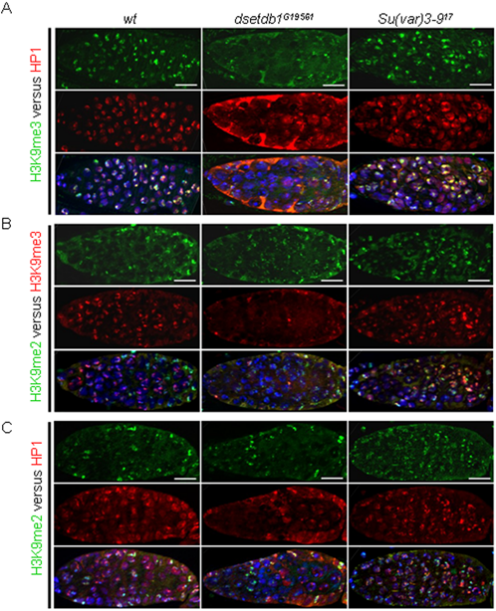
Relationship among di- and tri-methylated H3-K9 patterns, and HP1 pattern in *dsetdb1^G19561^* and *Su(var)3–9^17^* germaria. (A) H3K9me3 *versus* HP1 patterns. (B) H3K9me2 *versus* H3K9me3 patterns. (C) H3K9me2 *versus* HP1 patterns in wild-type (left panel), *dsetdb1^G19561^* (middle panel), and *Su(var)3–9^17^* (right panel) flies. Note that HP1 is absent from the inner germarium of the *dsetdb1^G19561^* mutant, from which H3K9me3 signals are absent (in A), whereas H3K9me2 signals remain unaltered (in C). Nuclei were counterstained with 4′,6-diamidino-2-phenylindole (DAPI; blue). Scale bars, 10 µm.

In a wild-type germarium, H3K9me3 signals overlapped with HP1 signals (Fig. 4A, left). However, lack of H3K9me3 in the *dsetdb1^G19561^* inner germarium led to failure of HP1 recruitment (middle). It was interesting that the mutant germaria have normal-looking H3K9me2 signals in the regions devoid of H3K9me3 (Fig. 4B, middle) and HP1 (Fig. 4C, middle) signals. This indicates that HP1 recruitment relies exclusively on H3K9me3 and not on H3K9me2, and that the presence of HKMTase(s) to synthesize the H3K9me3 substrate is essential for HP1 tethering to chromatin in modified histones. Western-blot analyses (Fig. 1E) showed that, in the *dsetdb1^G19561^* ovaries, H3K9me3 levels were obviously reduced, consistent with immunostaining observations, whereas the H3K9me2, H3K9me1, and HP1 levels did not seem to be significantly altered compared to the wild-type ovaries.

### dSETDB1 collaborates with SU(VAR)3-9 to establish H3K9me3 patterns in the outer germarium

**Figure 5:**
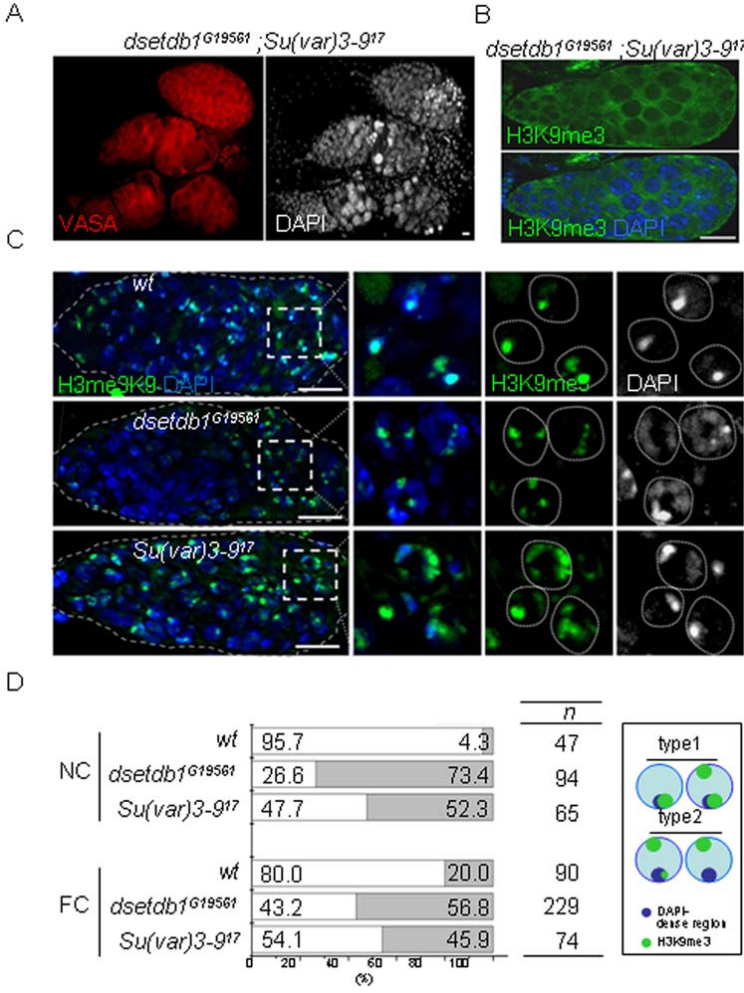
Disturbed trimethylated H3-K9 patterns in *dsetdb1^G19561^* and *Su(var)3–9^17^* germaria. (A) A *dsetdb1^G19561^; Su(var)3–9^17^* double mutant with gourd-shaped germaria. Note the VASA-positive cells accumulated in a mass in the posterior region of the germarium. (B) A *dsetdb1^G19561^; Su(var)3–9^17^* double mutant germarium lacked H3K9me3 signals. (C) H3K9me3 patterns in region-3 nurse cells of wild-type (upper panel), *dsetdb1^G19561^* (middle panel), and *Su(var)3–9^17^* germaria (lower panel). Boxed areas are enlarged into individual channels. Dotted lines indicate boundaries of nurse cell nuclei. Scale bars, 10 µm. (D) Frequency (%) of type-1 (white) and type-2 (gray) H3K9me3 patterns in nurse-cell (NC) nuclei and pre-follicle-cell (FC) nuclei (see text for classification). ‘n’ indicates the number of cells counted in each group. The schematic in the box illustrates the features of type-1 and type-2 cells graphically.

We found that the dSETDB1 signals were in cells throughout the germarium (Fig. 2C), but that only the ‘inner’ germarium cells were affected in the mutant germarium (Fig. 3B and C). Importantly, when GSC-derived cystocytes approached the region-3 and became nurse cells, they gained H3K9me3 signals in the *dsetdb1^G19561^* mutant (see arrows in Fig. 3C). This indicates that another H3-K9-specific methyltransferase might operate in the outer germarium, and the SU(VAR)3–9 is the most likely candidate. However, analysis of a *Su(var)3-9^17^* null mutant [45] showed that the whole germarium had solid H3K9me3 and HP1 signals (Fig. 4A). We hypothesized that *dsetdb1* might have a compensatory role in the *Su(var)3–9^17^* germarium, and therefore made a double mutant: *dsetdb1^G19561^; Su(var)3–9^17^*. The double mutants mostly had gourd-shaped germaria and defects in the budding off of new egg chambers; as a result, the double mutant had no developing egg chambers in the ovary (Fig. 5A). As shown in Fig. 5B, H3K9me3 signals were completely absent from the entire germarium in the double mutant. These results indicate that, in the outer germarium, dSETDB1 acts in concert with SU(VAR)3–9 to synthesize H3K9me3. Meanwhile, it is interesting to note that the *Drosophila dG9a* and *Su(var)3-9* double mutants *dG9a^13414^/dG9a^13414^ ;Su(var)3-9^06^/Su(var)3-9^06^* are fertile [25].

The disappearance of H3K9me3 signals from even the outer germarium in the *dsetdb1^G19561^;Su(var)3–9^17^* double mutant, but not in either of the single mutants, indicates that dSETDB1 or SU(VAR)3–9 alone can synthesize H3K9me3 signals in this area, but the resulting pattern may not be suitable for further development. We further explored the differences in H3K9me3 patterns produced by the two enzymes. It was repeatedly found that, compared with the wild type *Drosophila*, the *dsetdb1^G19561^* mutant contained relatively weak H3K9me3 signals, whereas the *Su(var)3–9^17^* mutant possessed stronger and more disperse signals (Fig. 5C). Detailed inspection allowed the H3K9me3 patterns of the region-3 nurse cells and pre-follicle cells in the germarium to be divided into two types on the basis of association (type-1) or non-association (type-2) of major H3K9me3 spots with DAPI-dense regions. In Fig. 5D, the type-2 cells account for 4% of wild-type nurse cells and for 73% and 52% of *dsetdb1^G19561^* and *Su(var)3–9^17^* nurse cells, respectively. Similar results were seen in pre-follicle cells, although the figures were less marked (20%, 57%, and 46% of wild-type, *dsetdb1^G19561^* and *Su(var)3–9^17^* cells, respectively). These results could indicate the question about how the absence of chromosomal H3K9me3 in the GSCs could be translated into the degeneration of egg chambers and result in female sterility. Our results show that both dSETDB1 and SU(VAR)3–9 are necessary to establish a proper H3K9me3 pattern in the outer germarium. Meanwhile, such aberrant localization of H3K9me3 signals was not found in a *dG9a* mutant [25], the mutant germarium of which exhibited normal-looking H3K9me3 and H3K9me2 patterns (Fig. S1).

### SU(VAR)3-9 solely functions in the developing egg chambers to trimethylate H3-K9 residues

**Figure 6:**
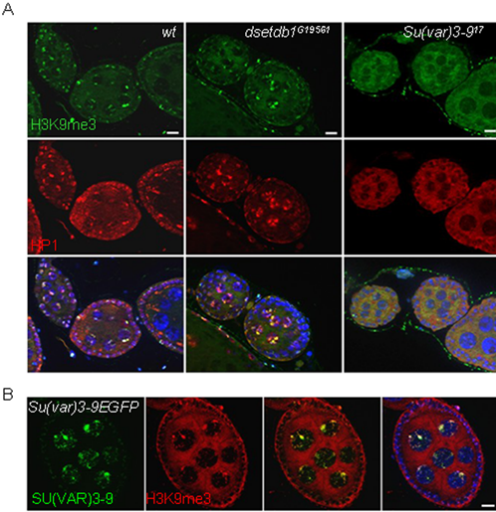
Lack of H3K9me3 signals in the *Su(var)3–9^17^* egg chambers. (A) H3K9me3 and HP1 expression patterns in wild-type, *dsetdb1^G19561^* and *Su(var)3–9^17^* egg chambers. The H3K9me3 signals colocalize with HP1 spots in wild-type and *dsetdb1^G19561^* cells, but neither H3K9me3 nor HP1 signals were detected in the *Su(var)3–9^17^* egg chambers. (B) Ectopic expression of SU(VAR)3-9-eGFP in the ovaries of transgenic flies, *{Gs[ry+,hs(Su(var)3-9 cDNA-EGFP)]}* [24], the expression of which was regulated by a heat-shock promoter. SU(VAR)3-9 signals in the nuclei of nurse cells and follicle cells co-localize with H3K9me3 signals in the developing egg chambers. Scale bars, 10 µm.

If the inner germarium was the primary region of H3K9me3 synthesis deficiency in the *dsetdb1^G19561^* ovary, the egg chambers were mainly affected in the *Su(var)3–9^17^* mutant ovary. Immunostaining of the *Su(var)3–9^17^* ovarioles showed that the egg chambers were totally devoid of H3K9me3 and HP1 signals (Fig. 6A, right panel), whereas egg chambers of the *dsetdb1^G19561^* mutants possessed strong signals for H3K9me3 and HP1 (middle). The RISH results showed that SU(VAR)3-9 functions in the developing egg chambers (Fig. 2A). Nonetheless, a complete absence of H3K9me3 signals from the developing egg chambers was unexpected because none of the H3-K9-specific methyltransferases, including *dsetdb1* and/or *dG9a,* replaced *Su(var)3-9* in the *Su(var)3–9^17^* egg chambers. We found that the ectopically expressed SU(VAR)3-9-eGFP signals were overlapped with H3K9me3 and with HP1 (data not shown) signals in developing egg chambers (Fig. 6B). The expression of *Su(var)3-9* transcripts was identified in the ovary, so it is likely that its translation products function and behave similarly to the GFP-tagged SU(VAR)3-9 in the developing egg chambers. These findings indicate that, during egg chamber growth, SU(VAR)3–9 solely maintains the H3K9me3 pattern inherited from the germarium, and that other H3-K9-specific methyltransferases involving dSETDB1 have nothing to do with this enzymatic process occurring in the egg chambers.

## Discussion

**Figure 7:**
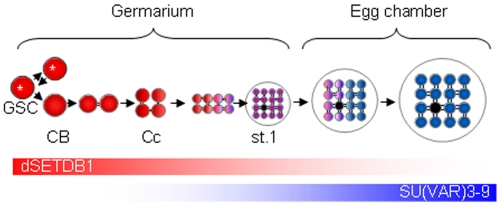
Illustration of differential allocation of dSETDB1 and SU(VAR)3-9 functions to GSC differentiation. The dSETDB1 (red) and the SU(VAR)3-9 (blue) proteins function in early and late GSC differentiation, respectively. The intensity of the color indicates the relative level of the activity. Both activities co-exist around region-3 germarium (stage-1 egg chamber, purple). Asterisks indicate self-renewing GSCs. For abbreviations, see the legend for Fig. 3.

Here, we showed that dSETDB1 is the only HKMTase responsible for the synthesis of H3K9me3 signals in the inner germarium where GSCs and their early descendants are found. When these vasa-positive cells move to region-3 germarium, the H3-K9 trimethylating task is transferred to a combination of dSETDB1 and SU(VAR)3–9, as both enzymes act cooperatively in all other somatic-type cells of the germarium. After the egg chamber buds off from the germarium, the trimethylation activity is now entirely the province of SU(VAR)3–9. The results, as illustrated in Fig. 7, disclose that the developmental program uses dSETDB1 first and then SU(VAR)3–9 during GSC differentiation, indicating that the two HKMTases perform distinct functions in these germ cells. The role of dSETDB1 in early GSC differentiation is presumably to “pre-mark” certain regions of chromatin, including the pericentric heterochromatin, with H3K9me3. The biochemical features of these pre-marked regions might be different from those of regions that are substrates of SU(VAR)3–9, and the pre-marked regions may be the platform on which incoming SU(VAR)3–9 further modulates the pre-methylated chromatin regions in later-developing VASA-positive cells. The functional significance of trimethylating, or “priming”, GSC chromatins with dSETDB1 is highlighted by the catastrophic ovarian phenotypes observed in, and the sterility of, the *dsetdb1* female homozygote. By contrast, although the egg chambers of the *Su(var)3–9^17^* mutant completely lacked H3K9me3 signals, which might be expected to result in a phenotype more severe than that of the *dsetdb1* mutant, the *Su(var)3–9^17^* mutant is capable of oogenesis, is able to lay eggs, and is fertile [45].

Meanwhile, the localization of both dSETDB1 and Su(var)3-9 at DAPI-dense heterochromatin does not necessarily mean that they target the same chromatin loci in early- and late-stage of oogenesis, respectively. The observation that dSETDB1, but not Su(VAR)3-9, is essential for Drosophila oogenesis provides a possibility that the two HKMTases may have different sets of target chromatin regions during oogenesis. It would be interesting to examine whether *dsetdb1* phenotypes could be rescued or not if exogenous Su(var)3-9 were expressed at high level in GSCs and their close derivatives.

In germ cells, dSETDB1 locates at DAPI-dense, pericentric heterochromatin (Fig. 2). This was unexpected because the mammalian counterpart, SETDB1/Eset, is known to have euchromatin-associated function [30]. Seum *et al.* recently reported that, in *Drosophila* polytene chromosomes, dSETDB1 locates at the fourth chromosome [27]. This fourth chromosome is known to be unusual as it has many characteristics of heterochromatic domains (such as a high-repeat density, no recombination and late replicating) and, at the same time, it shows features of euchromatin (such as being transcriptionally active and having a high gene density) [46]; in fact, many of the genes in the fourth chromosome are expressed during development [47]. These characteristics indicate that the banded regions of the fourth chromosome are different from pericentric heterochromatin, which highlights the peculiarity of dSETDB1 localization to DAPI-dense heterochromatin in the germarium.

The location of dSETDB1 at pericentric heterochromatin probably indicates that dSETDB1 participates, at a global level, in regulating chromosome organization and maintaining the chromosome integrity in the germ-lineages. This hypothesis is supported by the observation that the main H3K9me3 spots were displaced and went astray from DNA-dense heterochromatin regions in most region-3 cells of the *dsetdb1^G19561^* germarium (Fig. 5C). In addition, the egg chambers of the *dsetdb1^G19561^* mutant ovary that survived stage six were shown to have disorganized chromosomes in the nurse cell nuclei (Fig. S4). The nurse cell chromosomes of the stage-7 egg chambers in both wild-type and *Su(var)3–9^17^* ovaries were organized into bundles with well-developed, large nucleoli, whereas those in the *dsetdb1^G19561^* ovaries were simply scattered throughout the nucleoplasm without nucleolar regions; otherwise, all were stained positive in the TUNEL assay (Fig. 1Cf). HP1 was diffusely located in these nuclei of *dsetdb1^G19561^* mutant egg chambers but the HP1 mislocalization is unlikely to be the reason for the scattered chromosomes because the *Su(var)3–9^17^* egg chambers totally lacked HP1 but had nucleolar regions between bundles of chromosomes (Fig. S4). These results suggest that dSETDB1 has a role in coordinating the chromosomal integrity in the germ-cell lineages, and the loss of dSETDB1 function results in a dysregulation of chromosome organization.

In the ovary, the main type of methylation catalyzed by dSETDB1 is H3K9me3, in agreement with the results of a recent study [28]. In the *dsetdb1^G19561^* germarium, the loss of H3K9me3 was limited to the germ cells in the inner germarium. By contrast, in the salivary glands, dSETDB1 primarily synthesizes H3K9me2 [26], [27] at the fourth chromosome at which dSETDB1 itself localizes [27] together with POF [26]. Alterations in the H3K9me3 pattern and intensity were not detected in the polytene chromosomes in these studies. This means that dsetdb1 synthesizes either H3K9me2 or H3K9me3, depending on the type of cells in which it functions. By analogy with mammalian SETDB1/Eset [30], dSETDB1 can produce *in vitro* all the methylation types such as H3K9me1, H3K9me2 and H3K9me3. Under *in vivo* conditions, the specificity of SETDB1 activity and the resulting state of methylation depend on regulatory protein(s) associated with SETDB1/Eset. This is shown by the observation that a murine ATFa-associated factor (mAM) tightly associates with SETDB1 and facilitates the SETDB1-dependent conversion of H3K9me2 to H3K9me3 [38]. Therefore, the proteins that regulate SETDB1 activity determine the H3-K9 methylation state in certain tissue cells and at particular developmental stages, and this might be true for dSETDB1 in *Drosophila*.

Relating to likely dSETDB1-associated protein(s), a clue was provided by a recent study. A *dsetdb1* null mutant, *DmSetdb1^10.1a^* [27], dies at the late pupal stage, but it could be rescued to progress to the adult stage by expression of a truncated DmSETDB1^421–1,261^ transgene which was constructed by deleting the N-terminal 420 amino acids of the full-length dSETDB1. Of particular interest was the finding that the rescued females are sterile whereas the males are fertile, which is the same phenotype as seen with our *dsetdb1^G19561^* mutant. This rescue experiment indicates that the truncated DmSETDB1^421–1,261^ is enough for the null *DmSetdb1^10.1a^* mutants to survive the pupal stage, but is still insufficient to overcome the female sterility. This provides important clues about the tissue and substrate specificity of dSETDB1. This indicates that the N-terminal region (spanning 1–420 amino acids) of dSETDB1 is instrumental in female fertility. This region likely forms a functional domain that provides a binding site(s) for regulatory protein(s) that positions dSETDB1 at pericentric heterochromatin in the PGCs and GSC-derived cells instead of the fourth chromosomes, and preferentially synthesizes H3K9me3 instead of H3K9me2. Mammalian SETDB1/ESET is known to associate with several transcriptional regulators such as the ERG protein [39], mAM [38], KRAB-zinc-finger protein KAP1 [30], [31], and MBD1/MCAF1 [48], [49], [50]. It would be interesting to investigate the factor(s) that restricts dSETDB1 to the germ-cell lineages and favors H3K9me3 over H3K9me2 in the ovary.

At present, there is no information on SU(VAR)3-9 function during the *Drosophila* oogenesis. Because an antibody capable of immunocytochemically detecting SU(VAR)3-9 protein was unavailable, we used a transgenic line that expresses GFP-tagged SU(VAR)3-9 protein as an alternative [24]. It is clear that the ectopic expression pattern shown by the GFP-tagged SU(VAR)3-9 does not always reflect the pattern of endogenous SU(VAR)3-9. Nevertheless, if the SU(VAR)3-9-eGFP were expressed in a cell with endogenous SU(VAR)3-9, the SU(VAR)3-9-eGFP signals would be localized to wherever endogenous SU(VAR)3-9 is located. The results of RISH and RT-PCR analyses showed that *Su(var)3-9* is expressed in the ovarioles including the germarium and participates in oogenesis (Fig. 2A), and the egg chambers were shown to lack H3K9me3 in the *Su(var)3–9^17^* mutant flies (Fig. 6A). Therefore, a GFP-tagged SU(VAR)3-9 transgenic fly was used to determine the location of endogenous SU(VAR)3-9 from the ectopically expressed SU(VAR)3-9-eGFP signals in the ovarian cells, and the results showed that the SU(VAR)3-9-eGFP signals were overlapped with H3K9me3/HP1 signals in the egg chambers, indicating that endogenous SU(VAR)3-9 is responsible for H3K9me3 signals in developing egg chambers. The function of SU(VAR)3-9 in the germarium could also be deduced from the localization of SU(VAR)3-9-eGFP signals. In the inner germarium SU(VAR)3-9-eGFP signals were less co-localized with H3K9me3 signals than in the outer germarium (Fig. S2). Such a positioning of SU(VAR)3-9-eGFP in the germarium is in agreement with our prediction of endogenous SU(VAR)3-9 function in the outer germarium. Therefore, it is certain that SU(VAR)3-9 also has a role in the oogenesis. Despite its role as an influential epigenetic modifier, the SU(VAR)3-9 function during the oogenesis is likely to be dispensable because *Su(var)3-9* null mutant flies are fertile.

HP1 recognizes H3K9me2 and H3K9me3 [15], [40], [51]. In the polytene chromosomes of salivary glands, HP1 localizes at the chromocenter and chromosome 4 [52], which is in agreement with the pattern of H3K9me2, rather than H3K9me3 that is present at the core of the chromocenter [53]. Mutations in the *dsetdb1* gene abolish both H3K9me2 and HP1 signals from the fourth chromosome in the salivary glands [26], [27]. By contrast, the HP1 in the nuclei of both the germarium and the developing egg chambers mainly associates with H3K9me3 instead of H3K9me2. The *dsetdb1^G19561^* germarium and the *Su(var)3–9^17^* egg chambers have normal-looking H3K9me2 signals but lack H3K9me3, and their nuclei also lack HP1 signals (Fig. 4 and 6). These observations indicate that in some cells and tissues, HP1 binds either H3K9me2 or H3K9me3, and the preferred substrate depends on the HKMTase(s) itself that recruits and tethers HP1 to their sites of action.

In summary, we have demonstrated that *dsetdb1* is expressed, in a germ cell-specific manner, in the germarium; the germline stem cells and their early descendants reside in the anterior part of the germarium and both H3K9me3 and HP1 signals are abolished with mutations in the *dsetdb1* gene. In the GSC-derived cells, dSETDB1 trimethylates H3-K9 residues at pericentric heterochromatin, but this function is performed by SU(VAR)3–9 as germline cysts differentiate into egg chambers. Loss-of-function mutation in *Su(var)3–9* abolishes both H3K9me3 and HP1 signals in developing egg chambers. Both dSETDB1 and SU(VAR)3-9 collaborate in the region-3 germarium and a mutation in either of these genes causes localization of H3K9me3 away from DNA-dense regions in the region-3 cells. Our findings, therefore, indicate that *dsetdb1* and *Su(var)3-9* act sequentially to regulate chromosome organization in accordance with the differentiation of the germline-stem cells in *Drosophila*.

## Materials and Methods

### Fly stocks and genetics

Flies were cultured and kept at 25°C using standard methods. Wild-type control, *w^1118^*, the deficiency line *Df(2R)ED4065* (breakpoints 60C8-60E7), and *dG9a^13414^*, were obtained from the Bloomington Stock Center. The *dsetdb1^G19561^* mutant, which carries an EP element [33] inserted at a position 60 nucleotides downstream of the dsetdb1 translation initiation site, was obtained from GenExel. The *dsetdb1^G19561^* mutant alleles were balanced over *CyO,ActGFP* or *CyO,* to facilitate identification of homozygous individuals at larval and adult stages, respectively. To generate *detdb1* transgenic flies, a DNA fragment of ca. 2.7 kb, spanning the IP14531 sequence from 77–2759, and including only the regulatory domain, was first fused with a DsRed2 sequence and then cloned into the pUAST vector. Primer set used to amplify the *dsetdb1* region was 5′-CTA GAA TTC ATG TCT GGG CAG CCA ACA GCC C-3′ and 5′-GAT GGA TCC CGA GGG ATC AAC GGA GTA CTC GG-3′. The resulting pUAST-*dsetdb1*-ΔSET:DsRed2 construct was used for germline-mediated transformation. The strain *w^−^;ap^Xa^/CyO;TM3,Sb^1^* was the precursor of the double mutant with *dsetdb1^G19561^* and *Su(var)3–9^17^*. For *Su(var)3-9* expression, adult *pP{Gs[ry+,hs(Su(var)3-9 cDNA-EGFP)]}* females were heat shocked for 1 hr at 37°C and examined for GFP expression 6 hr later.

### Antibodies and immunohistochemistry

The antibodies against mono-methyl, di-methyl, and tri-methyl-H3-K9 (1∶200 for immunostaining; 1∶2000 for protein blots) were purchased from Upstate Biotechnology. The anti-HP1 antibody, C1A9 (1∶50 for immunostaining; 1∶500 for protein blots), and an anti-ORB antibody, 4H8, were purchased from the Developmental Studies Hybridoma Bank, University of Iowa. The anti-β-ACTIN and monoclonal anti-dimethyl-H3-K9 antibodies were from Abcam. The anti-histone H3 (1∶2000 for protein blots) and anti-VASA (used at 1∶100) antibodies were from Santa Cruz Biotechnology. An anti-dSETDB1 polyclonal antibody (1∶50 for immunostaining; 1∶5,000 for protein blots) was raised using a synthetic peptide, N-YFDGTTCSRGKDKGC-C, as an immunogen. Alexa-488-labeled dSETDB1 antibody was made using Zenon rabbit IgG labeling kit (Molecular Probes). Briefly, 2 µg of purified dSETDB1 antibody was incubated with the Zenon Rabbit IgG labeling reagent for 10 minutes at room temperature, then 5 µl of the Zenon blocking reagent was mixed to the mixture and incubated for 10 minutes at room temperature. Fluorescence-conjugated (1∶200, Molecular Probes) or horseradish peroxidase (HRP)-conjugated (1∶5000, Santa Cruz Biotechnology) secondary antibodies were used to visualize signals. Images were taken using a Carl Zeiss Axiovert 200M fluorescence microscope equipped with an ApoTome.

### TUNEL assay

TUNEL (terminal deoxynucleotidyl transferase (TdT)-mediated dUTP nick end labeling) assay was carried out using the *In Situ* Cell Death Detection Kit (Roche). Ovaries were dissected in PBS, fixed for 20 minutes in 4% formaldehyde and rinsed three times in PBT (0.1% Tween-20 in PBS). Ovaries were then incubated in TUNEL reaction mixture (including FITC-conjugated modified nucleotides and TdT) for 1 hour at 37°C with humidity. The samples were rinsed in PBT and stained with DAPI. TUNEL signal was visualized directly under a FITC filter. Images were taken using a Zeiss AxioVert 200M fluorescence microscopy with ApoTome.

### RT-PCR

Total RNAs from designated tissues or organ were extracted by TRIZOL (Invitrogen) according to the manufacturer's instructions. Two micrograms of total RNAs were used for synthesis of first-strand cDNA using oligo-d(T) primer and Superscript II reverse transcriptase (Invitrogen) for 1 hour at 42°C. The *dsetdb1* sequence was amplified with *dsetdb1* specific primer set, 5′-TTT ACC AGG TGC TCC GAA AGT CT-3′ and 5′-TCT TCG ATG AGC TGC AGC TTG TT-3′ in a ABI 9700 thermal Cycler (ABI) in the cycling condition of 95°C for 30 s, 55°C for 30 s, and 72°C for 30 s. For amplifying a 206-bp fragment of *rp49* cDNA sequence, we used primers, 5′-AGT CGG ATC GAT ATG CTA AG-3′ and 5′-TTC TCT TGA GAA CGC AGG TA-3′. 600-bp and 240-bp fragments of *dsetdb1* sequence were individually amplified using primers, 5′-TTC CTG AAA AAG ATG AAA AGA CCA A-3′ and 5′- TCT TCG ATG AGC TGC AGC TTG TT -3′, and 5′- TTT ACC AGG TGC TCC GAA AGT CT -3′ and 5′- TCT TCG ATG AGC TGC AGC TTG TT -3′, respectively. For amplification of a 271-bp *Su(var)3-9* sequence, primers 5′-CAA GCG GTC GAA AAA TAA CAT GGG-3′ and 5′-TGC CTC CAG CTG CTT CTC AAG CT-3′ were used.

### Whole mount mRNA *in situ* hybridization

For whole mount mRNA *in situ* hybridization, ovaries obtained from wild-type females were dissected, fixed in 4% formaldehyde and 10% DMSO in PBS, and incubated with proteinase K. Extracted ovaries were prehybridized in hybridization buffer (50% formamide, 5×SSC and 100 µg/ml salmon sperm DNA, 50 µg/ml heparin and 0.1% Tween-20) and hybridized overnight with digoxygenin (DIG, Roche) labeled RNA probe at 45°C. After several rinses with PBT (0.1% Tween-20 in PBS), ovaries were incubated for 2 hr at room temperature or overnight at 4°C with AP-conjugated anti-DIG antibody before color visualization using the NBT/BCIP reaction (Roche). Ovaries were dehydrated in 100% ethanol for at least 10 min, and cleared and mounted in 50% glycerol. Images were taken using an Olympus BX60 microscopy.

DIG-labeled sense and anti-sense RNA probes were made according to the manufacturer's instructions. Primer sets for preparation of *dsetdb1* template (spanning IP14531 from 2201–2896) was 5′-TAC ACC AAG GAG ATG GAG TC-3′ and 5′-CAG ATT CAG ATG AAC ACC CT-3′. For *Su(var)3-9* (spanning NM_079633.2 from 633-1184), we used primers, 5′-GCT GCG AGG AAC ATG ATG TGG AC-3′ and 5′-TAC AAA TTG GGC CCG CTT GAT TTG-3′. For *dG9a*, a primer set we used was 5′-GAT GGG AAA ACG AAA GCT TTA AAA C-3′ and 5′-TCC CCT AAT GAA TCC TCG CAG GG-3′. The PCR product was cloned into pGEM-T easy vector (Promega). These plasmids were individually used for preparing sense and antisense RNA probes using the T7 and Sp6 promoters in the vector, respectively.

## Supporting Information

Figure S1 H3-K9 methylation patterns in the dG9a13414 germarium. (A) Whole-mount mRNA in situ hybridization using a RNA probes for dG9a. Arrows indicate the germarium. dG9a transcripts are abundantly accumulated in the stage-10 egg chambers but there is no clear evidence of expression at the germarium and earlier-stage egg chambers. (B) H3K9me2 patterns in the dG9a 13414 germarium. (C) Double-staining for H3K9me3 and HP1 in the dG9a13414 germarium. There were no alterations in methylated H3-K9 patterns in the dG9a 13414 germarium. (D) Frequencies of type-1 and type-2 H3K9me3 patterns in stage-1 nurse cells and follicle cells in the region-2b/3 dG9a 13414 germarium (see text for type classification). NC, nurse cell; FC, follicle cell. Scale bars, 10 µm.
(10.04 MB TIF)

Figure S2 Ectopic expression of SU(VAR)3-9-eGFP in the germarium. GFP expression was examined in the germarium of SU(VAR)3-9-eGFP-expressing transgenic flies, *{Gs[ry+,hs(Su(var)3-9 cDNA-EGFP)]}* [24], in which SU(VAR)3-9 expression was regulated by a heat-shock promoter. In general, SU(VAR)3-9-eGFP signals were not localized with H3K9me3 signals in the germarium. Ectopically expressed SU(VAR)3-9-eGFP signals are less co-localized with H3K9me3 signals in the anterior part of the germarium (Cc, cystocytes), where endogenous SU(VAR)3-9 is assumed to be absent under normal conditions, than in the posterior part of the germarium. NC, nurse cell; FC, pre-follicle cell. Scale bars, 10 µm.
(6.36 MB TIF)

Figure S3 dSETDB1 locates in DAPI-dense nuclear region of would-be oocytes of the growing egg chambers. (A) Strong dSETDB1 signals in the would-be oocytes (arrows). dSETDB1 locating at the karyosome of would-be oocyte that is stained for a marker, ORB, for maturing oocytes. dSETDB1 signals in nuclei of nurse cells and follicle cells (arrows) of a stage-4 egg chamber. (B) Exclusion of SU(VAR)3-9-eGFP signal from the karyosome of a would-be oocyte in an egg chamber. SU(VAR)3-9-eGFP was ectopically expressed in the egg chamber. dSETDB1, but not the SU(VAR)3-9-eGFP, localizes to the karyosome. Dashed circle in (A) indicates the nucleus of a would-be oocyte. Scale bars, 10 µm.
(4.55 MB TIF)

Figure S4 Disturbed chromosome organization in the nurse-cell nuclei of *dsetdb1^G19561^* stage-7 egg chamber. Nurse-cell chromosomes of the stage-7 egg chambers are organized into bundles with well-developed, large nucleoli in both wild-type and *Su(var)3-9^17^* ovaries. By contrast, chromosomes at the same stage in the *dsetdb1^G19561^* ovary were distributed throughout the nucleoplasm and there was not sufficient space for a normal-looking nucleolus when compared with the wild type ovaries. Note that HP1 was diffusely localized in these nuclei of *dsetdb1^G19561^* mutant egg chambers, and absent from the *Su(var)3-9^17^* egg chambers.
(4.00 MB TIF)
