# Supplementary figures and images for: dSETDB1 and SU(VAR)3–9 Sequentially Function during Germline-Stem Cell Differentiation in Drosophila melanogaster
Source: PLoS One. 2008 May 21;3(5):e2234. doi: 10.1371/journal.pone.0002234 (PMC2377335; doi:10.1371/journal.pone.0002234)

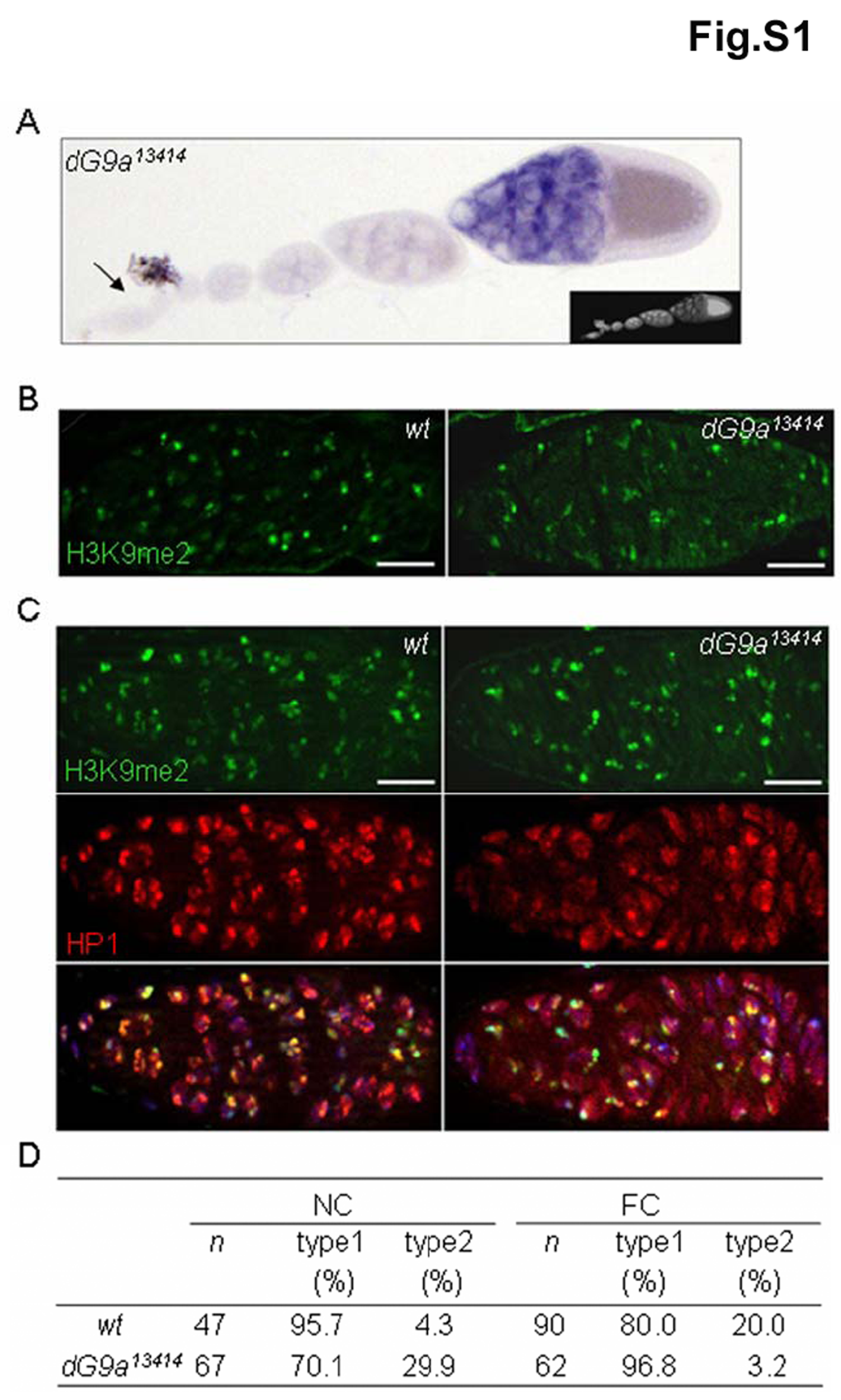

Supplement: Figure S1 — H3-K9 methylation patterns in the dG9a13414 germarium. (A) Whole-mount mRNA in situ hybridization using a RNA probes for dG9a. Arrows indicate the germarium. dG9a transcripts are abundantly accumulated in the stage-10 egg chambers but there is no clear evidence of expression at the germarium and earlier-stage egg chambers. (B) H3K9me2 patterns in the dG9a 13414 germarium. (C) Double-staining for H3K9me3 and HP1 in the dG9a13414 germarium. There were no alterations in methylated H3-K9 patterns in the dG9a 13414 germarium. (D) Frequencies of type-1 and type-2 H3K9me3 patterns in stage-1 nurse cells and follicle cells in the region-2b/3 dG9a 13414 germarium (see text for type classification). NC, nurse cell; FC, follicle cell. Scale bars, 10 µm. (10.04 MB TIF) [file pone.0002234.s001.tif]

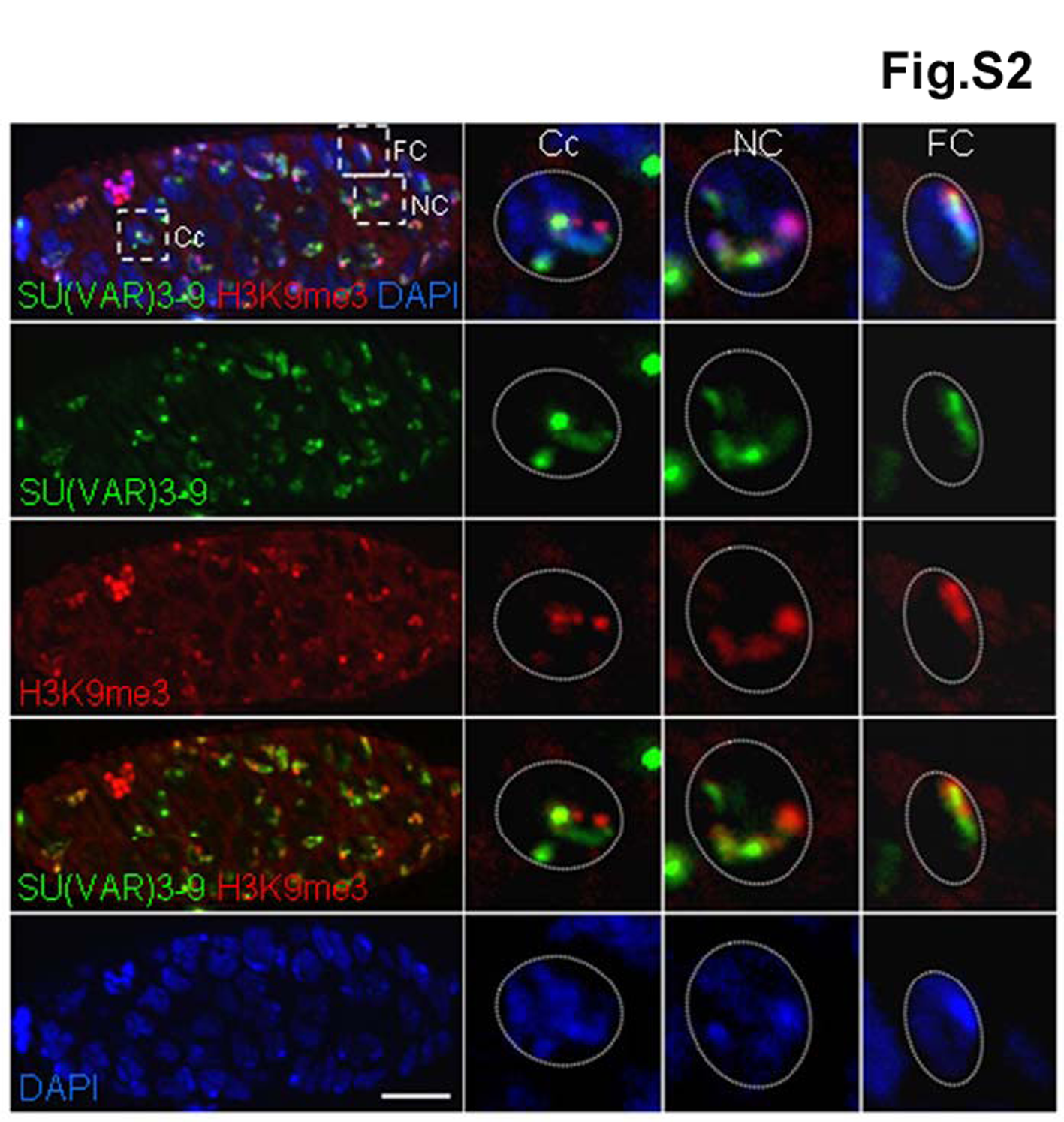

Supplement: Figure S2 — Ectopic expression of SU(VAR)3-9-eGFP in the germarium. GFP expression was examined in the germarium of SU(VAR)3-9-eGFP-expressing transgenic flies, {Gs[ry+,hs(Su(var)3-9 cDNA-EGFP)]} [24], in which SU(VAR)3-9 expression was regulated by a heat-shock promoter. In general, SU(VAR)3-9-eGFP signals were not localized with H3K9me3 signals in the germarium. Ectopically expressed SU(VAR)3-9-eGFP signals are less co-localized with H3K9me3 signals in the anterior part of the germarium (Cc, cystocytes), where endogenous SU(VAR)3-9 is assumed to be absent under normal conditions, than in the posterior part of the germarium. NC, nurse cell; FC, pre-follicle cell. Scale bars, 10 µm. (6.36 MB TIF) [file pone.0002234.s002.tif]

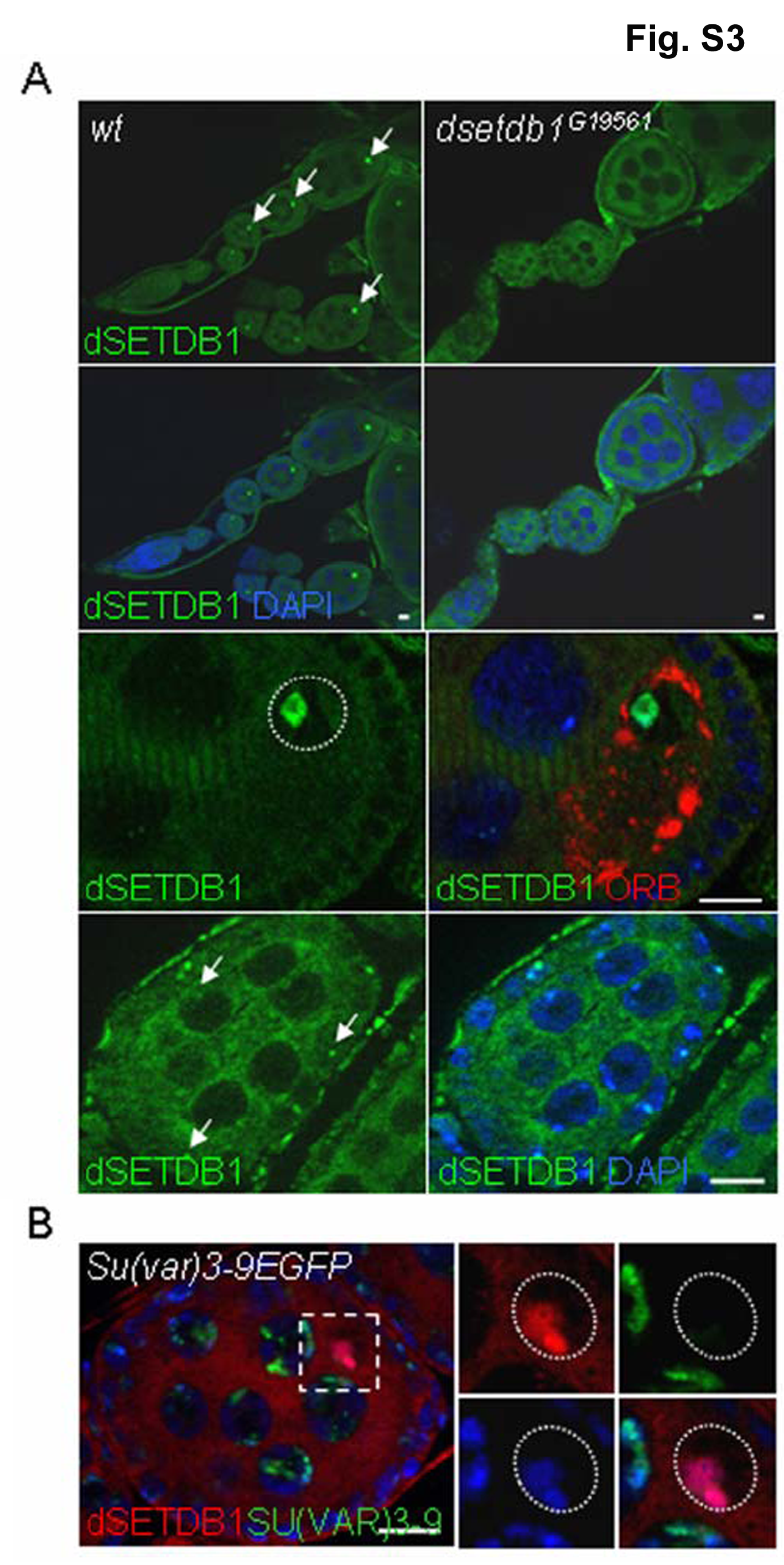

Supplement: Figure S3 — dSETDB1 locates in DAPI-dense nuclear region of would-be oocytes of the growing egg chambers. (A) Strong dSETDB1 signals in the would-be oocytes (arrows). dSETDB1 locating at the karyosome of would-be oocyte that is stained for a marker, ORB, for maturing oocytes. dSETDB1 signals in nuclei of nurse cells and follicle cells (arrows) of a stage-4 egg chamber. (B) Exclusion of SU(VAR)3-9-eGFP signal from the karyosome of a would-be oocyte in an egg chamber. SU(VAR)3-9-eGFP was ectopically expressed in the egg chamber. dSETDB1, but not the SU(VAR)3-9-eGFP, localizes to the karyosome. Dashed circle in (A) indicates the nucleus of a would-be oocyte. Scale bars, 10 µm. (4.55 MB TIF) [file pone.0002234.s003.tif]

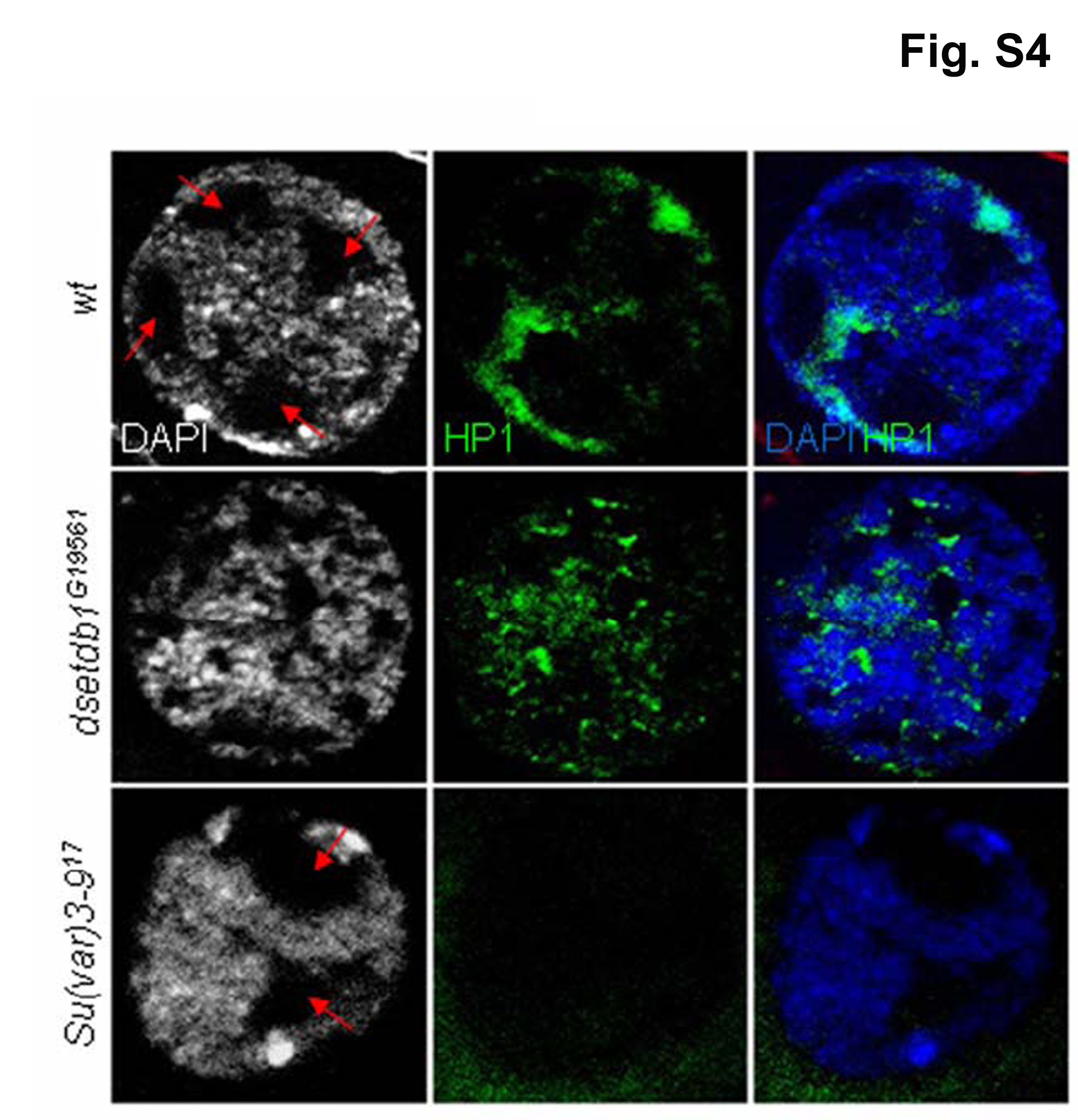

Supplement: Figure S4 — Disturbed chromosome organization in the nurse-cell nuclei of dsetdb1G19561 stage-7 egg chamber. Nurse-cell chromosomes of the stage-7 egg chambers are organized into bundles with well-developed, large nucleoli in both wild-type and Su(var)3-917 ovaries. By contrast, chromosomes at the same stage in the dsetdb1G19561 ovary were distributed throughout the nucleoplasm and there was not sufficient space for a normal-looking nucleolus when compared with the wild type ovaries. Note that HP1 was diffusely localized in these nuclei of dsetdb1G19561 mutant egg chambers, and absent from the Su(var)3-917 egg chambers. (4.00 MB TIF) [file pone.0002234.s004.tif]
